# Risk Analysis of a Fuel Storage Terminal Using HAZOP and FTA

**DOI:** 10.3390/ijerph14070705

**Published:** 2017-06-30

**Authors:** José Luis Fuentes-Bargues, Mª Carmen González-Cruz, Cristina González-Gaya, Mª Piedad Baixauli-Pérez

**Affiliations:** 1 Departamento de Proyectos de Ingeniería, Universitat Politècnica de València, Camino de Vera s/n, 46022 Valencia, Spain; mcgonzal@dpi.upv.es; 2 Departamento de Ingeniería de Construcción y Fabricación, ETSII, UNED, C/Ciudad Universitaria s/n, 28040 Madrid, Spain; cgonzalez@ind.uned.es; 3 Universitat de València, Avda. de la Universidad s/n, 46100 Valencia, Spain; mabaipe@alumni.uv.es

**Keywords:** risk, HAZard and OPerability analysis (HAZOP), Fault Tree Analysis (FTA), fuel, storage

## Abstract

The size and complexity of industrial chemical plants, together with the nature of the products handled, means that an analysis and control of the risks involved is required. This paper presents a methodology for risk analysis in chemical and allied industries that is based on a combination of HAZard and OPerability analysis (HAZOP) and a quantitative analysis of the most relevant risks through the development of fault trees, fault tree analysis (FTA). Results from FTA allow prioritizing the preventive and corrective measures to minimize the probability of failure. An analysis of a case study is performed; it consists in the terminal for unloading chemical and petroleum products, and the fuel storage facilities of two companies, in the port of Valencia (Spain). HAZOP analysis shows that loading and unloading areas are the most sensitive areas of the plant and where the most significant danger is a fuel spill. FTA analysis indicates that the most likely event is a fuel spill in tank truck loading area. A sensitivity analysis from the FTA results show the importance of the human factor in all sequences of the possible accidents, so it should be mandatory to improve the training of the staff of the plants.

## 1. Introduction

Technological and social development has led to an increase in the size and complexity of chemical plants. At the same time, the existence of such plants and the transport of their products involve certain risks that need to be controlled and minimised [1,2].

Risk is understood as the possibility that someone or something is adversely affected by a hazard [3], while danger is defined as any unsafe situation or potential source of an undesirable and damaging event [4]. Other definitions of risk are the measure of the severity of a hazard [5], or the measure of the probability and severity of adverse effects [6].

In recent decades, interest in the safety of chemical industrial plants has greatly increased [2,7]. This has led to the development of a scientific discipline known as process safety that focuses on the prevention of fires, explosions, and accidental chemical releases in chemical processing facilities [8]. This discipline has as objective to improve prevention in the facilities, learning from accidents and from continuous analysis of the production process.

Directive 2012/18/EU (or Seveso III) [9] defines as a serious accident an event (such as a major leak, fire, or explosion) resulting from an uncontrolled process during the operation of any plant and producing a serious danger, whether immediate or delayed, to human health or the environment, inside or outside the plant, and involving one or more hazardous substances. Examples of serious accidents in industrial processes include: Flixborough in Britain (1974), Seveso in Italy (1976), Bhopal in India (1984), Enschede in the Netherlands (2000), Toulouse in France (2001) and Buncefield in Britain (2005) [10,11,12,13,14,15]. In Spain, examples include an accident at the Repsol refinery in Puertollano (2003) in which an explosion in a gas storage area killed nine workers and injured many others, as well as causing property damage.

The complexity and severity of accidents at these plants requires the implementation of risk management systems. The ISO 31000: 2010 [16] standard defines risk management as “coordinated activities to manage and control an organisation with regard to risk” and comprises the following steps: communication and consultation, establishing the context, risk assessment (identification, analysis, and evaluation), risk treatment, monitoring, and review.

The purpose of this article is to show the procedure for risk analysis in chemical and allied industries that is based on a combination of HAZard and OPerability analysis (HAZOP) and a quantitative analysis of the most relevant risks through the development of fault trees, fault tree analysis (FTA). HAZOP can identify possible fault root causes and their consequences and FTA develops fault propagation pathways and provides a quantitative probability importance ranking of fault causes. These results can guide the decision making of management staff to mitigate or avoid potential process hazards. This working method is applied to a case study consisting of the terminal for unloading chemical and petroleum products, and the fuel storage facilities of two companies, in the port of Valencia (Spain).

This paper is organized as follows. Section 1 introduces the theme. Section 2 introduces the main data of the chemical industry in Spain and the framework for risk assessment process of major accidents. Section 3 introduces the methodology. Section 4 details a case study with the HAZOP and FTA analysis. Section 5 presents the conclusions. Appendix A, Appendix B, Appendix C and Appendix D present complementary documentation of case study.

## 2. The Chemical Industry in Spain and Serious Accidents

### 2.1. The Chemical Industry in Spain

Turnover of the chemical industry in Spain totalled €56.39 billion in 2014, representing 12.4% of industrial Gross Domestic Product (GDP) [17] and making the industry the fourth largest after the food, transport and metal industries. This is also the second largest sector of the Spanish economy in terms of exports with 58.1% of sales going abroad.

The largest concentration of chemical companies is found in Catalonia with 43% of total turnover, followed by Andalusia (12.7%) and Madrid (13.5%). The Valencian Community is in fourth place with €4.88 billion or 8.4% of total turnover. The chemical sector employed 191,100 people in 2008, a figure that has fallen to around 174,600 in recent years because of the economic crisis [17].

The Spanish Chemical Industry Federation (FEIQUE) in its 2015 annual report on industrial accidents in the chemical sector [18] noted that the frequency index was 3.44 (the index frequency represents the number of accidents for every million hours worked). Compared with data published by the Ministry of Employment in 2015, this index is lower than the industrial sector index (5.03) and the construction sector index (6.59). The severity index for the sector was 0.12 (the severity index represents the number of days lost per 1000 working hours), which reflects the great importance that is given to safety in the Spanish chemical industry.

### 2.2. The Regulatory Framework

The disastrous accident at Seveso (Italy) in 1976 led to European Union legislation intended to prevent accidents in certain industries using hazardous substances and thus limit the impact on employees, the general population, and on the environment. The resulting standard was Directive 82/501/EEC [19] better known as Seveso I. This regulatory framework established that a manufacturer company which used in their process hazardous substances listed in the Appendix A or stored hazardous substances listed in the Appendix B, or both, must develop (among other documents) interior and exterior protection and emergency plans that include risk assessment.

During the implementation of Seveso I, there were more than 130 serious accidents in Europe and new risks appeared due to technological advances. Consequently, the European Commission introduced Directive 96/82/EC (called Directive Seveso II) [20] in 1996. This directive classified plants into “not affected”, “low risk” and “high risk” according to the quantities of dangerous substances present. Seveso II was revised in Directive 2012/18/EU or Seveso III [9] with the aim of increasing levels of protection for people, property, and the environment.

In Spain, in 2016, according to data from the Directorate General for Civil Defence [21], there were 422 high risk plants subject to the Seveso directive and 470 low risk plants. The geographical distribution is similar to that for turnover: Catalonia was first with 101 high risk plants (23.9%), Andalusia with 70 (16.6%), the Valencian Community with 39 (9.2%) and the Basque Country with 28 (6.6%).

According to a study by Planas et al. [2], there have been 89 accidents in Spain since the beginning of the twentieth century. Some 44% of these accidents occurred during transport, the most serious accident occurring at *Los Alfaques* campsite in July 1978 where 217 people died. The second major source of accidents were processing areas (19%); and the third source were storage areas. Explosions occurred in 49% of accidents, leaks in 37% and fires in 24%.

The chemical industry has implemented improvements in process safety and environmental protection with four strategies: inherent safer design; risk assessment processes; use of instrumented safety systems; and the implementation of safety management systems. In the risk assessment process, the HAZOP method is the technique most used to identify risks [2]. HAZOP studies evolved from the Imperial Chemical Industries (ICI) as a “Critical Examination” technique formulated in the mid-1960s. One decade later, HAZOP was published formally as a disciplined procedure to identify deviations to the process industries by Kletz in 1978 [22], and some publications [23], corporate guidelines, standards (IEC 61882 [24]) and national guidance notes (*Nota Técnica Prevención* (NTP) 238 [25]) were developed after.

## 3. Methodology

Risk assessment is the process of identifying, analysing, and evaluating the hazard posed by an industrial plant and the main aim is the prevention and mitigation of accidents in potentially hazardous facilities [26,27].

The phase of hazard identification is the process in which hazards are identified and recorded. The analysis phase involves developing an understanding of the hazard and providing information for evaluation. The evaluation phase involves comparing the estimated hazard levels with predefined criteria to define the importance of the level of hazard and decide whether it is necessary to address the hazard—as well as the most appropriate strategies and methods of hazard treatment [8].

Choosing the appropriate risk assessment techniques is a difficult decision that will depend on factors such as the complexity of the problem, the methods for analysis of the amount of information available, the need for quantitative data, and available resources [28]. Often, authors combine some techniques with the purpose of blending, i.e., to take advantage of the strengths of each method whilst compensating for their weaknesses.

In this paper, the methodology used is based on the combination of HAZOP analysis and a quantitative analysis of the most relevant hazards by FTA. HAZOP is a qualitative technique that carries out a structured analysis of the process and allows identifying the deviations that may take place with regard to the intended functioning, as well as their causes and consequences. HAZOP does not try to provide quantitative results but, in many situations, it is necessary to rank the identified hazards, mainly to prioritize the actions to mitigate them because this decision depends of the risk level. For this purpose, HAZOP is combined with other techniques; in these cases, quantitative techniques such as FTA. It can identify the potential causes and the ways of failure and can assess quantitatively the probability of development of the accident. The blending of the two techniques was defined as positive because minimize the uncertainty [29,30,31].

There are many examples of blending HAZOP and FTA in the literature: Demichela et al. [32] developed the Recursive Operability Analysis (ROA), linking HAZOP results and FTA development; Cozzani et al. [33] developed a specific methodological approach to analyse the risk from hazardous materials in marshalling yards; Casamirra et al. [34] integrated HAZOP, FTA and Failure Mode and Effect Analysis (FMEA) to assess the safety of a hydrogen refuelling station; and Kim et al. [35] combined HAZOP and FTA to carry out safety assessment of hydrogen fuelling stations at Korea.

**Figure 1:**
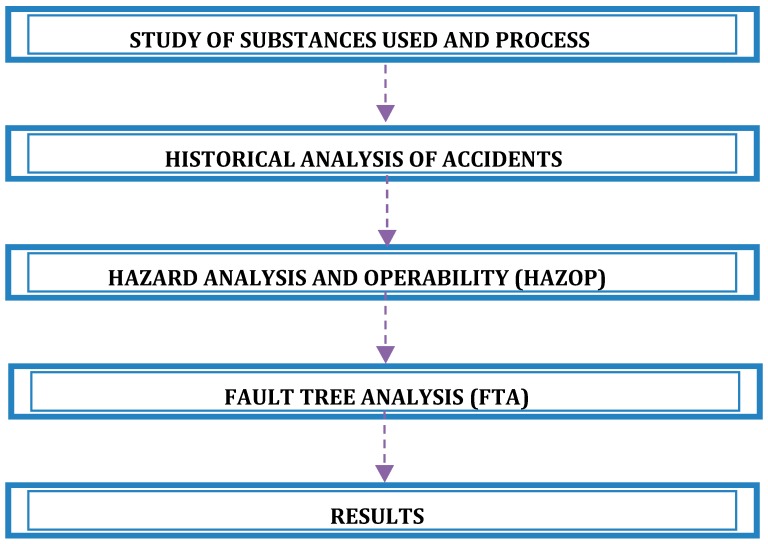
Methodology of study.

The methodology (Figure 1) begins with a detailed study of the industrial process and substances used. Subsequently, an historical analysis of accidents is made—which is the study and analysis of accidents in similar plants to identify risk and causes. This stage is performed by referring to specialised scientific publications and literature review. With this available information, a HAZOP analysis is conducted. After the HAZOP sessions, the possible fault causes and consequences of the given deviations from the design are identified. These data allow, according to the criteria of the HAZOP team, identifying the initiating events, modelling the fault propagation process, and finally building the fault tree analysis. Subsequently a quantitative analysis is performed and results obtained rank risks and allow prioritizing the corrective and/or preventive measures.

### 3.1. HAZOP Method

The HAZOP technique [36] is a structured and systematic examination of a product, process, or procedure—or an existing or planned system. This is a qualitative technique based on the use of guide words (Table 1) that question how design intent or operating conditions may fail to be achieved at each step of the design process or technique. The guide words must always be appropriately selected to the process which is analysed and additional guide words can be used.

**Figure 2:**
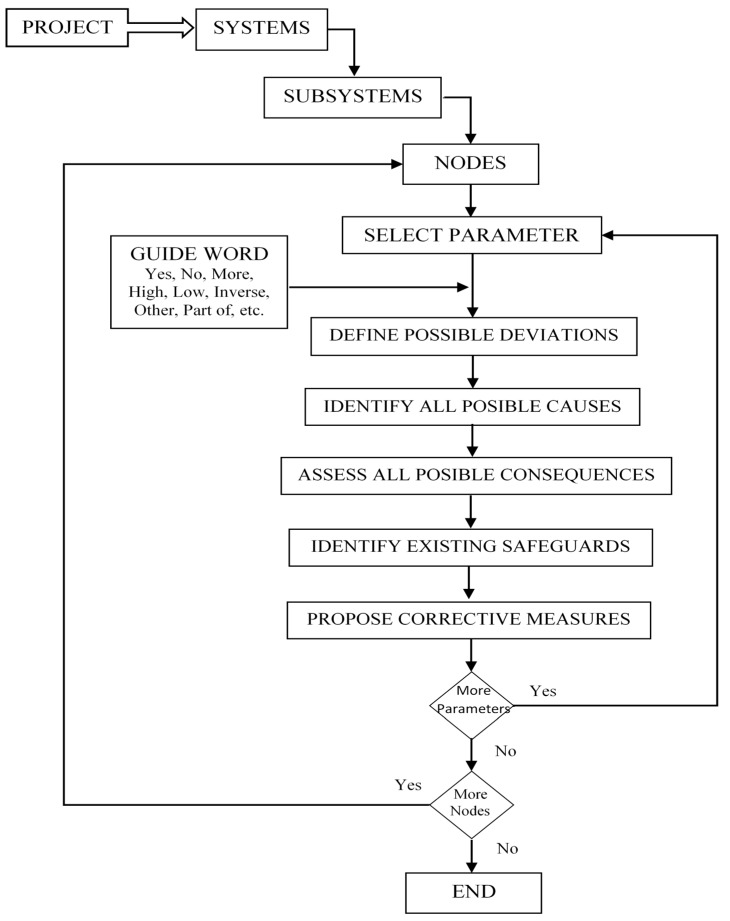
HAZard and OPerability analysis (HAZOP) process.

This technique is applied by a multidisciplinary team during a series of meetings where work areas and operations are defined—and each of the variables that influence the process are applied to the guide to verify the operating conditions and detect design errors or potentially abnormal operating conditions (Figure 2).

### 3.2. Fault Tree Analysis

FTA is a technique to identify and analyse factors that may contribute to an unwanted specified event (called the “top or main event”). Causal effects are identified deductively and organised in a logical manner and shown using a tree diagram that describes the causal factors and their logical relationships (Table 2) with respect to the top event.

A fault tree can be used qualitatively to identify potential causes and the ways in which failure (the top event) occurs or quantitatively, or both, to calculate the probability of the top event from the probabilities of causal events.

The stages for the application of this technique are:

(1) Define the top event.
(2) Construction of the fault tree: From the top event, the possible immediate causes of the failure modes are established and it is possible to identify how these failures can occur at basic levels or in basic events.
(3) Qualitative evaluation: The aim to find the minimum set of faults, establishing a mathematical formulation from the relationships established in the fault tree. To achieve this, the “OR” gates are replaced by the “+” sign (not addition but a union of conjunctions) and the gates “AND” by the “x” sign (equivalent to the intersection of conjunctions). Boolean algebra is used.
(4) Quantitative evaluation: From the frequency of failure of basic events, the probable frequency of an accident is calculated (if it occurs) as well as the most critical fault routes (i.e., the most probable among combinations of susceptible events that may cause the top event). Quantitative evaluation enables a complete risk analysis before implementing and prioritising actions to improve the safety and reliability of the system under study. A complementary sensitivity analysis can be performed to check the effect of the basic events in the global risk assessment. These data allow prioritizing the preventive measures and the efforts of the risk control process.

## 4. Application to a Case Study: The Chemical Terminal at the Port of Valencia

The application of the methodology is performed for the jetty and pipe work of the chemical terminal, as well as the connected storage facilities, at the Port of Valencia. These storage facilities are owned by two companies: Terminales Portuarias SL (TEPSA) and Petróleos de Valencia SA (PTROVAL) [38,39]. Both companies work in the reception, storage, loading, and distribution of liquid products—divided into two groups: chemicals and oil.

### 4.1. Identification of Products Handled

TEPSA stores and distributes gasoline, diesel, methanol, and other chemicals in smaller amounts. PTROVAL (owned by Galp Energía) stores and distributes gasoline, diesel, and kerosene. The four substances (petrol, diesel, methanol, and kerosene) are hazardous substances according to Schedule I of Royal Decree 1254/1999 [40] and the large volumes handled mean that the plant is considered high risk under the Seveso classification. Such high-risk plants are required to conduct a risk analysis.

### 4.2. Historical Analysis of Accidents

Chang et al. [41] performed a study of storage tank accidents in industrial facilities between 1960 and 2003. They collected and reviewed 242 tank accidents, 207 belonging to crude oil, oil products (fuel oil, diesel, kerosene, lubricants), gasoline/naphtha and petrochemicals products. The main causes of tanks accidents were in order of importance: lightning (33.1%), maintenance (13.2%), operational error (12.0%), equipment failure (7.9%), sabotage (7.4%), crack/rupture (7.0%), leaks and line rupture (6.2%) and static electricity (3.3%).

Person and Lönnermark [10] listed 479 fires involving hydrocarbon storage tanks between 1951 and 2003. Based on this work, Hailwood et al. [11] identified 21 tank explosions followed by a fire.

In a specific study of risk assessment for Liquefied Natural Gas (LNG) terminals, Aneziris et al. [42] identified the initiating events of accidents of LNG terminals. They divided the LNG terminals in five areas: LNG tanks, unloading section (from ship to tank), send-out section, condenser and outlet pipeline. 

In tanks section, the main initiating events are boil-off removal malfunction (during unloading or during storage), a high temperature in LNG (when coming from ship), an excess of external heat in storage tank area, an overfilling of the tank, a rollover during unloading or during storage, an inadvertent starting of additional compressors, a continuation of uploading beyond lower safety level and an increase of send out rate from tank. In unloading section, the main initiating events are an excess external heat in jetty area, a water hammer in loading arm (due to inadvertent valve closure), an inadequate cooling of lading arm and high winds during uploading.

In Appendix A, a list of well documented past accidents has been extracted from reports and works available in the literature. The list includes accidents in petroleum and LNG product storage facilities [12,13,43,44].

The origins of these accidents were leaks or spills (9), explosions (7) and fire (6). Leakage (in the form of liquid) is the most common source of major accidents—leading to fires and explosions that may cause other leaks, thus lengthening the accidental chain. The possible consequences of leakage depend on the flammability and toxicity of the leaked liquids and the environmental conditions in which the leak occurs. 

Seventeen of the cases originated in storage tanks, two in tanker ships, one in pipes, one in a steam boiler of a LNG plant and in one case there was no specific origin.

Factors that may cause an accident are grouped into general and specific. Among the general causes are those that are: external to the plant, human behaviour, mechanical failure, failure caused by impact, violent reactions; instrumentation failure, and failure of services. These general causes include a number of specific causes provided by details of specific accidents. Note that a single accident can occur for more than one general cause, and a general cause may be the result of more than one specific cause. The recorded data on the general causes of accidents shows that the cause was human behaviour in ten cases, instrumentation failure on four occasions, electrostatic spark on two occasions, mechanical failure in two occasions, unknown causes on two occasions, and two accidents were caused respectively by mechanical impact failure and external causes respectively.

Ignition sources provided the energy needed for the combustion of a flammable mixture. These sources can be thermal, electrical, mechanical and chemical. Data shows that in seven accidents the cause was electrical, in three the cause was welding during maintenance works, mechanical in three cases, thermal in two cases, and unknown in seven cases.

### 4.3. HAZOP Analysis

**Figure 3:**
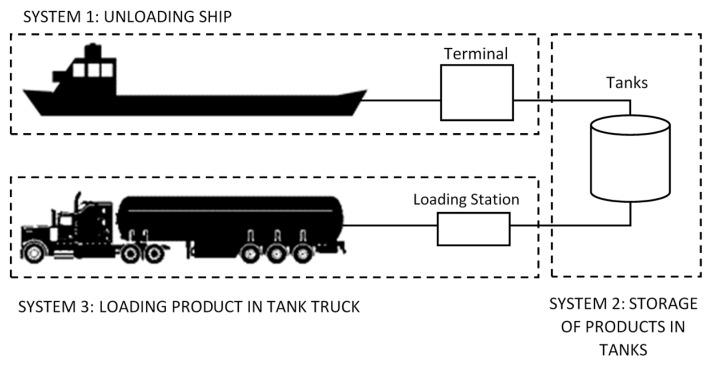
Three areas of activity.

The Valencian plant is divided into three systems (Figure 3) that correspond to the three activities of the companies: unloading, storage, and loading for distribution.

These three systems are divided into six sub-systems and these again are divided into specific points or nodes that correspond to the sequence of operational steps in the plant (Table 3). Table 4 shows guide words and parameters used in the HAZOP analysis and Table 5 shows the result of the HAZOP analysis for node 2.1.1 (opening tank valves) and some variables of node 2.1.2 (filling tank).

As a result of this analysis, it can be seen that, in the areas for loading and unloading liquid products (Systems 1 and 3), the greatest danger is the possibility of an uncontrolled spill. The occurrence of this event is closely linked to the effectiveness of the staff responsible for handling the tasks. Relative to System 2, the risk of a fuel loss in the pipelines and leakage or fuel loss in the storage tanks is noteworthy. The latter event could be caused by overfilling or a partial rupture of the tank. Special attention must be given to such events because they can cause fires and explosions that may have more serious consequences for the plant and its staff.

### 4.4. Fault Tree Analysis (FTA)

By using HAZOP analysis, four events were extracted for analysis using the fault tree technique. These events or top events were:

▪ Top event (1): Fuel spill in ship-terminal unloading area.
▪ Top event (2): Fuel leak in pipelines.
▪ Top event (3): Fuel spill in storage tank.
▪ Top event (4): Fuel spill in tank truck loading area.

**Figure 4:**
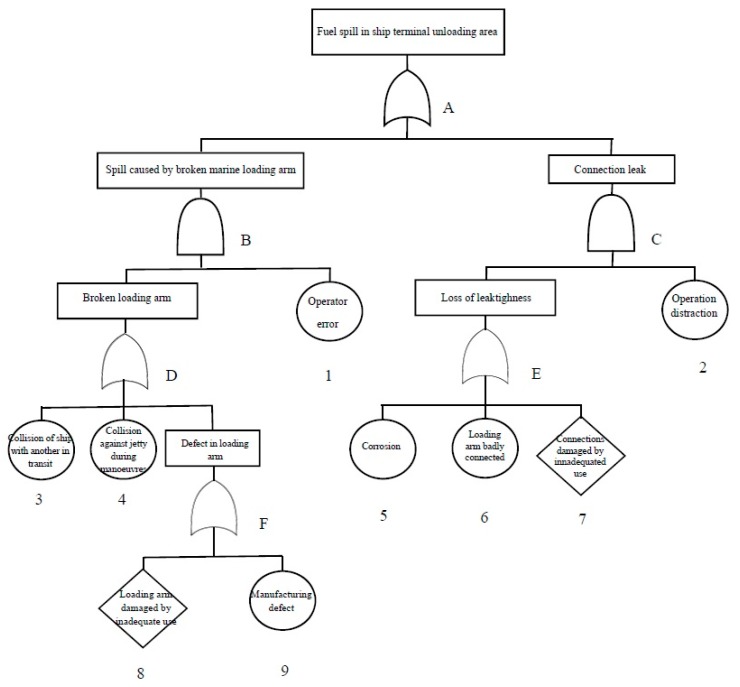
Top event fault tree (1).

The faults and relationships for each top event have been identified and a logical combination of incidents has been deduced that can trigger unwanted events. In this way, each tree contains information about how the combination of certain faults leads to overall failure (Figure 4). Appendix B presents the fault trees of the other top events. 

Once the fault trees have been made, the mathematical expressions are defined ant the probability values are calculated according to the Boolean algebra related to FTA (Table 6 and Table 7).

From these equations and data on the frequency of failures of basic events, a quantitative assessment of the trees enables a calculation of the probability of the occurrence of the top event (year^−1^). The procedure for calculating the top event (1) is shown in Table 7. In the four analysed top events, some 19 basic events are defined and fault frequencies were determined using data from the Spanish National Institute on Health and Safety at Work [45] and research on fuel storage [12,41,46,47]. In the Appendix C similar tables are developed for the others top events.

In Table 8, the results of failure frequency for each of the top events and their ways of failure are presented. A column called “Importance” has been added in order to show the importance of the failure frequency of the events (and also of their ways of failure) developed through the fault tree technique. The results indicate that the top event (4) “Fuel spill in tank truck loading area” has a failure rate of 1.7 events/year, i.e., 85% of the events developed through the fault tree technique. There are two ways a top event (4) can be generated: the first is via a “connection leak” with an importance of 80.28% and the second is via “leak caused by broken hose” which accounts for 5.02% of importance. If the basic events are analysed, the main causes for a connection leak are a bad hose connection and a response failure following the detection of an emergency (incorrect staff response, failure of the acoustic alarm, or seizure of the manual closure valve). 

The next most significant source of risk for the overall failure sequence is “connection leakage” in the top event (1) “Fuel spill in ship-terminal unloading area” (with a failure frequency of 0.17 events/year). This event occurs following a loss of product (caused by a bad connection of the loading arm or damaged parts) together with human error. The probability of occurrence is low since it is one of the most complex operations and involves very strict protocols.

**Figure 5:**
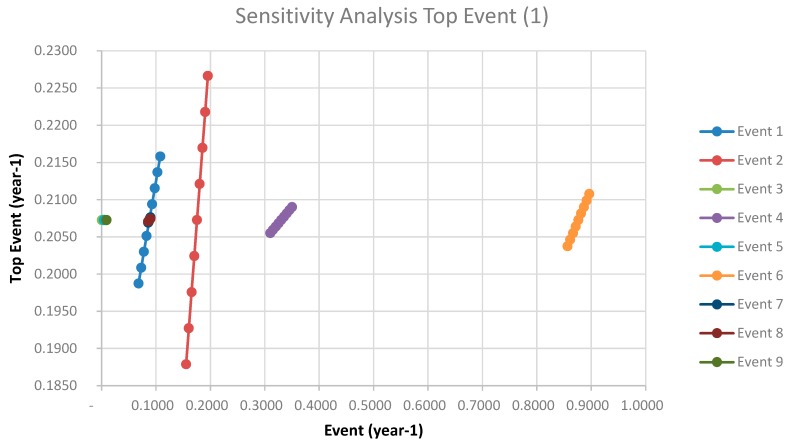
Sensitivity Analysis for the Top event (1).

A sensitivity analysis has been performed (see Appendix D) in order to check the effect of the basic events in the global risk assessment. In the top event (1) (Table 9 and Figure 5), the basics events with more influence in the sequence of the accident are in order of importance: operator distracted, operator failure, badly connecting loading arm and collision against jetty during manoeuvres. In the top event (2) are corrosion, operator distracted and with the same importance vehicles collision and fatigue defect. In the top event (3) are operator failure and with equal importance the failure of the sensor level and the failure of response of the shut-off valve. In the top event (4) are hose incorrectly connected, after with equal importance, the acoustic signal failure and the sticking of the manual shut-off valve, and in the fourth level the operator failures. These results show the importance in all the sequences of accident of the failure or distraction of the operators, so it should be mandatory a plan for training the staff of the plants. Planning of the maintenance actions of the facility must take into account both the general results from the risk assessment and the results from the sensitivity analysis.

## 5. Conclusions

In this paper, a methodology that combines HAZOP analysis and FTA is used. HAZOP analysis identifies the risks and their possible causes and consequences. FTA, based on the HAZOP analysis, represents the fault propagation pathways and produces a qualitative and quantitative assessment of the sequences of events that can lead to accidents or serious failures. Results from FTA allow prioritizing the preventive and corrective measures in order to minimize the probability of failure. 

An analysis of case study about a fuel storage terminal is performed. HAZOP analysis shows that loading and unloading areas are the most sensitive areas of the plant and where the most significant danger is a fuel spill—tasks that can produce such an event are closely supervised by staff. Tasks related to transferring fuel from ships to tanks and storage tanks are the most automated and so the influence of personnel is reduced—although the consequences are more serious if an accident occurs. FTA analysis indicates that the most likely event is “Fuel spill in tank truck loading area” and the sequence of events that would most likely cause such an event is a “connection leakage” caused by improper hose connection and a failure of emergency systems. A sensitivity analysis of the FTA results shows the importance of the human behaviour in all sequences of the possible accidents. A slight increase or decrease of the frequency of failure of human operations generate an important increase or decrease, respectively, of the frequency of failure of the top event, so corporation’s prevention plans must increase the training of the staff, develop of automatic control measures and develop or improve control procedures to check the human operations.

In future research, we will apply a similar analysis to other type of plant, as LNG plants or storage of chemical products at a process plant, in order to improve the use of the combined method and to compare results from the risk assessments. In this way, we will build a database of HAZOP cases and FTA analysis and could improve the maintenance plans of the various types of plants.

## Figures and Tables

**Table 1:** HAZard and OPerability analysis (HAZOP) guide word method. Source: ISO 31010: 2011 [27].

Guide Word	Meaning	Example of Deviation
NO	Absence of the variable to which it applies	No flow in line
LESS	Quantitative reduction	Less flow
MORE	Quantitative increase	Higher temperature
OTHER	Partial or total replacement	Other substances were added
INVERSE	Opposite function to design intention	Return flow
PART OF	Qualitative decline. Only part of what should happen occurs	Part of volume required by recipe was added
IN ADDITION	Qualitative increase. More is produced than intended	In addition of the amount of water of the process was added

**Table 2:** Symbols used in fault trees. Source: ISO 31.010:2011 [27] and Vesely et al. [37].

Symbol	Meaning	Description
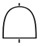	Logic gate AND	The output event happens only if all input events happen
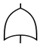	Logic gate OR	The output event occurs if any of the input events happen
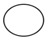	Basic event	Failure of a component that has no identifiable primary cause. It is the highest level of detail in the tree
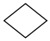	Undeveloped event	Failure of a component with a primary cause undeveloped because of lack of information
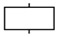	Intermediate event	A fault event that occurs because of one or more antecedents causes acting through logic gates

**Table 3:** Systems, subsystems, and nodes for HAZOP analysis.

System		Sub-System		Nodes	
1	Unloading ship	1.1	Connection ship terminal	1.1.1	Docking ship at terminal
				1.1.2	Extension of marine loading arm
				1.1.3	Joining of marine arm and manifold
		1.2	Transfer to tanks	1.2.1	Opening of valves
				1.2.2	Product movement
				1.2.3	Closure of valves
				1.2.4	Cleaning of tubes
2	Storage of product in tanks	2.1	Filling tanks	2.1.1	Opening tank valves
				2.1.2	Filling tank
				2.1.3	Closing tank valves
		2.2	Product storage	2.2.1	Product storage
3	Loading product in tank truck	3.1	Arrival at loading station	3.1.1	Positioning of tank truck
				3.1.2	Flexible hose connection to tank truck
		3.2	Transfer from tanks	3.2.1	Opening tank truck valves
				3.2.2	Transfer and filling of tank
				3.2.3	Valve closure

**Table 4:** Guide Words and Parameters used in the HAZOP analysis.

ID System	ID Sub-System	ID Nodes	Guide Word	Parameter
1	1.1	1.1.1	Wrong/More	Mooring/Speed
		1.1.2	Other/No/Less	Direction/Movement/Safety
		1.1.3	Other/No/No/Less	Element/Connection/Electrical Isolation /Safety
	1.2	1.2.1	No/Less/More/More/More	Flow/Flow/Speed/Static Electricity/Corrosion
		1.2.2	More-Less/Less/Less/More/Yes/More	Pressure/Maintenance/Flow/Static Electricity/Collision/Corrosion
		1.2.3	Yes/More/More-Less/More/More	Flow/Speed/Pressure/Static Electricity/Corrosion
		1.2.4	No/Less	Cleaning/Pressure
2	2.1	2.1.1	No/Less/More/More/More	Flow/Flow/Speed/Static Electricity/Corrosion
		2.1.2	More/More	Level/Static electricity
		2.1.3	Yes/More/More-Less/More/More	Flow/Speed/Pressure/Static Electricity/Corrosion
	2.2	2.2.1	Yes/More/More/Less	Flammability/Corrosion/Pressure/Maintenance
3	3.1	3.1.1	Wrong/Wrong/Different	Entry into the loading bay/Manoeuvrability at the loading bay/Loading position
		3.1.2	Less/Less	Connection/Safety
	3.2	3.2.1	No/Less/More/More/More	Flow/Flow/Speed/Static Electricity/Corrosion
		3.2.2	More/No/Yes/More/Less	Level/Connection/Stop filled/Static Electricity/Safety
		3.2.3	Yes /More/More-Less/More/More	Flow/Speed/Pressure/Static Electricity/Corrosion

ID: Identity.

**Table 5:** Example of HAZOP analysis for nodes 2.1.1 and 2.1.2.

**Node 2.1.1: Tank Opening Valves**				**System 2: Product Storage in Tank**	
				**Sub-System 2.1: Filling Tank**	
**Guide Word**	**Variable**	**Deviation**	**Possible Causes**	**Possible Consequences**	**Comments and Corrective Measures**
More	Static electricity	Accumulation of static electricity than expected	Circulation of liquid in the valve. Bad earth grounding.	Possible risk of explosion if difference in electrical potential occur.	The faster the speed of flow, the greater charge generated. Valves and flanges that are completely painted should be conductively bridged and earthed.
More	Corrosion	More corrosion of materials than expected	Exposure to corrosive environment. Attack of impurities at points with imperfections or fatigue. Lack of maintenance.	Uniform deterioration of surface of valve (general corrosion). Reduction in the useful life (weakening).	The best way to avoid corrosion is to select the most resistant alloy for the valve– depending on the corrosive nature of the fluids. When damage is minor and possible to repair the body of the valve—at least provisionally—with a metal weld or with epoxy resin (for low pressures and temperatures).
**Node 2.1.2: Filling Tank**				**System 2: Product Storage in Tank**	
				**Sub-System 2.1: Filling Tank**	
**Guide Word**	**Variable**	**Deviation**	**Possible Causes**	**Possible Consequences**	**Comments and Corrective Measures**
More	Level	More level than expected (overfill)	Faulty level sensor. Incorrect valve setting. Supervisor failure to recognise problems.	Product over flow. Spill of liquid down external tank walls. Formation of inflammable atmosphere as fuel hits floor. If source of ignition exists there is serious risk of explosion and/or pool fire with chain reaction to affect nearby tanks.	Activate tank vents to reduce or stop emissions of vapour. Staff training. Renewal of level sensors. Verification of state of all valves. Automatic level alarms as operator activated redundant safety devices. Use of indicators that measure volume to avoid confusion with specific weight. Spill containment berm system should have a capacity greater than the tanks (including safety percentage).
More	Static electricity	Accumulation of static electricity than expected	Liquid projected by jet. Liquid enters tank being filled. Movement of liquid in tank causing turbulence and splashing.	Production of electrostatic sparks with sufficient energy to cause ignition. Generation of extremely serious fires and/or explosions.	As a safety measure, it is recommended that the filling tube is always below the liquid surface level (meaning that it reaches the floor), or if not possible, the flow should be reduced. Fluids should slide along the walls of tanks so that charges can dissipate through the earthed protective coverings. Speed of fluid should not exceed 7 m/s. Air humidity should be around 60%.

**Table 6:** Qualitative evaluation of top event (1).

Top Event (1) Fuel Spill Ship-Terminal Unloading Area	
Equations System	Boolean Equation
A = B + C	A = (3 × 1) + (4 × 1) + (8 × 1) + (9 × 1) + (5 × 2) + (6 × 2) + (7 × 2)
B = D × 1	
C = E × 2	
D = 3 + 4 + F	
E = 5 + 6 + 7	
F = 8 + 9	

**Table 7:** Top event failure frequencies (1).

Top Event (1) Fuel Spill in Ship-Terminal Area		
Basic Event	Description	Failure Frequency (year^−1^)
1	Operator failure	8.8 × 10^−2^
2	Operator distracted	1.8 × 10^−1^
3	Ship collision with another in transit	6.0 × 10^−4^
4	Manoeuvring collision against jetty	3.3 × 10^−1^
5	Corrosion	4.4 × 10^−3^
6	Badly connected loading arm	8.8 × 10^−1^
7	Damaged connection caused by inadequate use	8.8 × 10^−2^
8	Loading arm damaged by inadequate use	8.8 × 10^−2^
9	Manufacturing defect	8.8 × 10^−3^
B	Leakage caused by broken loading arm	3.7 × 10^−2^
C	Connection leak	1.7 × 10^−1^
A = B + C	Top event (1)	2.1 × 10^−1^

**Table 8:** Results of quantitative analysis.

Description	Frequency of Failure (year^−1^)	Importance (%)
Top event (1): Fuel spill in ship-terminal unloading area	0.21	10.54
Leakage caused by broken loading arm	0.037	2.00
Connection leak	0.17	8.53
Top event (2): Fuel leak in pipelines	0.0081	0.41
Breakage caused by cracking	0.0061	0.31
Undetected leak	0.0020	0.10
Top event (3): Leak in storage tank	0.075	3.76
Overfilling	0.063	3.16
Loss of leak tightness	0.012	0.60
Top event (4): Fuel spill in tank truck loading area	1.7	85.29
Leak caused by broken hose	0.085	5.02
Connection leak	1.6	80.28

**Table 9:** Sensitivity Analysis for the Top event (1).

Top Event (1) Fuel Spill Ship-Terminal Unloading Area							
Equations System				A = B + C = (3 × 1) + (4 × 1) + (8 × 1) + (9 × 1) + (5 × 2) + (6 × 2) + (7 × 2)			
Event 1	B	C	A	Event 2	B	C	A
0.1077	0.0460	0.1698	0.2158	0.1953	0.0375	0.1892	0.2266
0.1027	0.0439	0.1698	0.2137	0.1903	0.0375	0.1843	0.2218
0.0977	0.0417	0.1698	0.2115	0.1853	0.0375	0.1795	0.2170
0.0927	0.0396	0.1698	0.2094	0.1803	0.0375	0.1747	0.2121
0.0877	0.0375	0.1698	0.2073	0.1753	0.0375	0.1698	0.2073
0.0827	0.0353	0.1698	0.2051	0.1703	0.0375	0.1650	0.2024
0.0777	0.0332	0.1698	0.2030	0.1653	0.0375	0.1601	0.1976
0.0727	0.0310	0.1698	0.2009	0.1603	0.0375	0.1553	0.1927
0.0677	0.0289	0.1698	0.1987	0.1553	0.0375	0.1504	0.1879
Event 3	B	C	A	Event 4	B	C	A
0.0008	0.0375	0.1698	0.2073	0.3502	0.0392	0.1698	0.2090
0.0008	0.0375	0.1698	0.2073	0.3452	0.0388	0.1698	0.2086
0.0007	0.0375	0.1698	0.2073	0.3402	0.0383	0.1698	0.2081
0.0007	0.0375	0.1698	0.2073	0.3352	0.0379	0.1698	0.2077
0.0006	0.0375	0.1698	0.2073	0.3302	0.0375	0.1698	0.2073
0.0006	0.0374	0.1698	0.2073	0.3252	0.0370	0.1698	0.2068
0.0005	0.0374	0.1698	0.2073	0.3202	0.0366	0.1698	0.2064
0.0005	0.0374	0.1698	0.2073	0.3152	0.0361	0.1698	0.2060
0.0004	0.0374	0.1698	0.2073	0.3102	0.0357	0.1698	0.2055
Event 5	B	C	A	Event 6	B	C	A
0.0046	0.0375	0.1699	0.2073	0.8966	0.0375	0.1733	0.2108
0.0045	0.0375	0.1698	0.2073	0.8916	0.0375	0.1724	0.2099
0.0045	0.0375	0.1698	0.2073	0.8866	0.0375	0.1716	0.2090
0.0044	0.0375	0.1698	0.2073	0.8816	0.0375	0.1707	0.2081
0.0044	0.0375	0.1698	0.2073	0.8766	0.0375	0.1698	0.2073
0.0043	0.0375	0.1698	0.2073	0.8716	0.0375	0.1689	0.2064
0.0043	0.0375	0.1698	0.2073	0.8666	0.0375	0.1681	0.2055
0.0042	0.0375	0.1698	0.2072	0.8616	0.0375	0.1672	0.2046
0.0042	0.0375	0.1698	0.2072	0.8566	0.0375	0.1663	0.2038
Event 7	B	C	A	Event 8	B	C	A
0.0897	0.0375	0.1702	0.2076	0.0897	0.0376	0.1698	0.2074
0.0892	0.0375	0.1701	0.2075	0.0892	0.0376	0.1698	0.2074
0.0887	0.0375	0.1700	0.2074	0.0887	0.0375	0.1698	0.2074
0.0882	0.0375	0.1699	0.2074	0.0882	0.0375	0.1698	0.2073
0.0877	0.0375	0.1698	0.2073	0.0877	0.0375	0.1698	0.2073
0.0872	0.0375	0.1697	0.2072	0.0872	0.0374	0.1698	0.2072
0.0867	0.0375	0.1696	0.2071	0.0867	0.0374	0.1698	0.2072
0.0862	0.0375	0.1696	0.2070	0.0862	0.0373	0.1698	0.2071
0.0857	0.0375	0.1695	0.2069	0.0857	0.0373	0.1698	0.2071
Event 9	B	C	A				
0.0090	0.0375	0.1698	0.2073				
0.0089	0.0375	0.1698	0.2073				
0.0089	0.0375	0.1698	0.2073				
0.0088	0.0375	0.1698	0.2073				
0.0088	0.0375	0.1698	0.2073				
0.0087	0.0374	0.1698	0.2073				
0.0087	0.0374	0.1698	0.2073				
0.0086	0.0374	0.1698	0.2073				
0.0086	0.0374	0.1698	0.2073				

**Figure A1:**
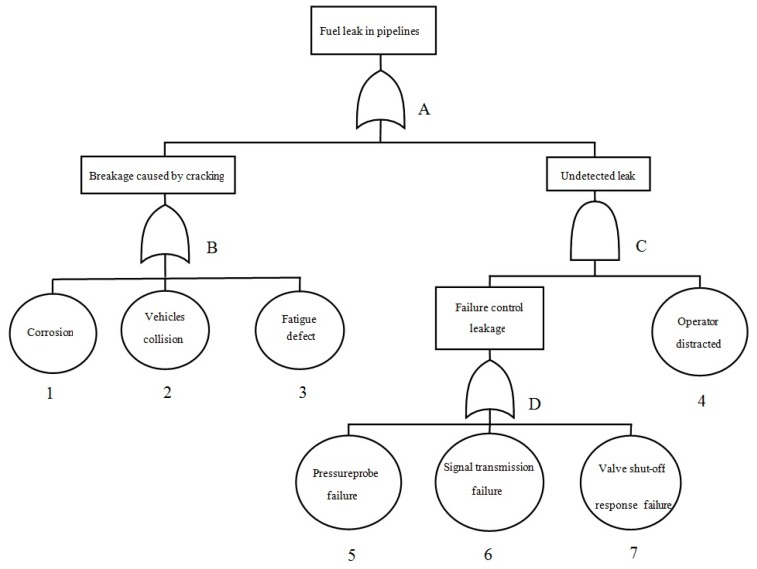
Top event fault tree (2).

**Figure A2:**
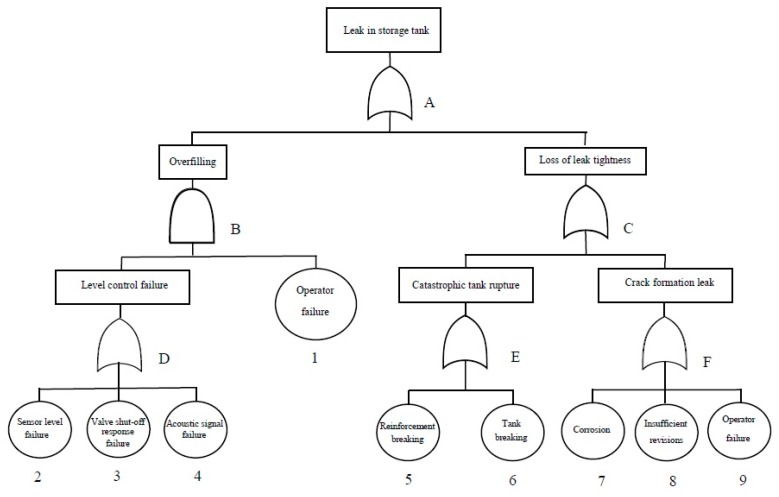
Top event fault tree (3).

**Figure A3:**
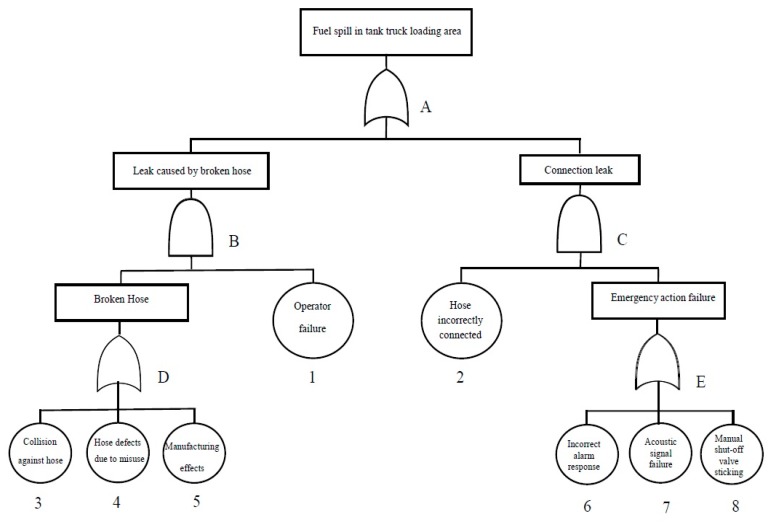
Top event fault tree (4).

**Figure A4:**
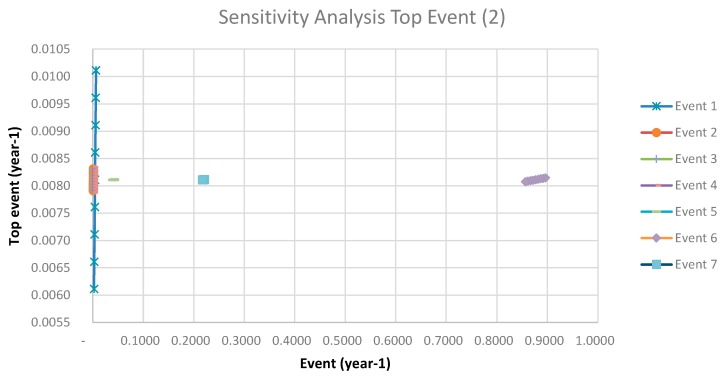
Sensitivity Analysis for the Top event (2). Events 1 to 7.

**Figure A5:**
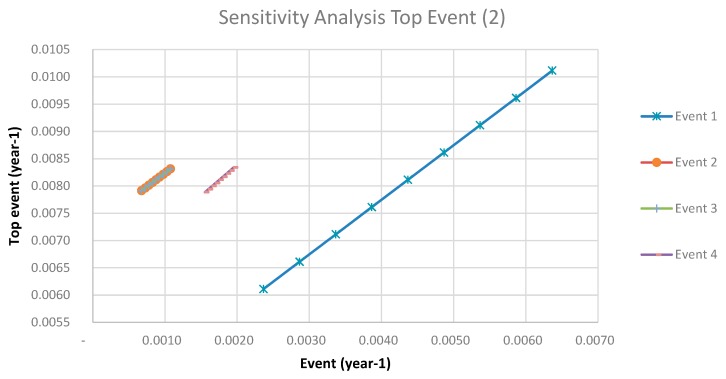
Sensitivity Analysis for the Top event (2). Events 1 to 4.

**Figure A6:**
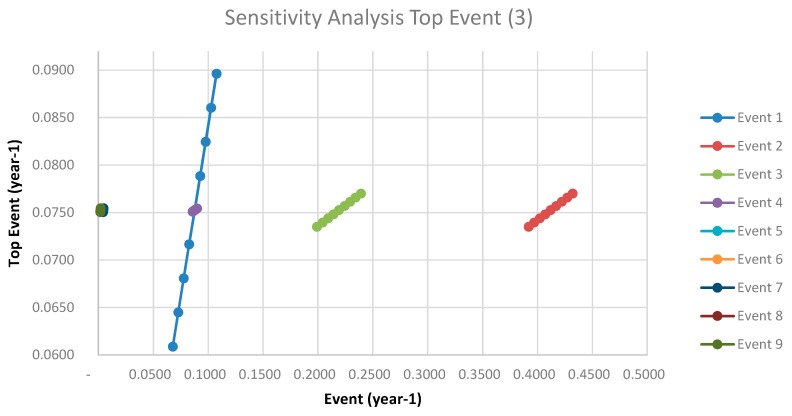
Sensitivity Analysis for the Top event (3).

**Figure A7:**
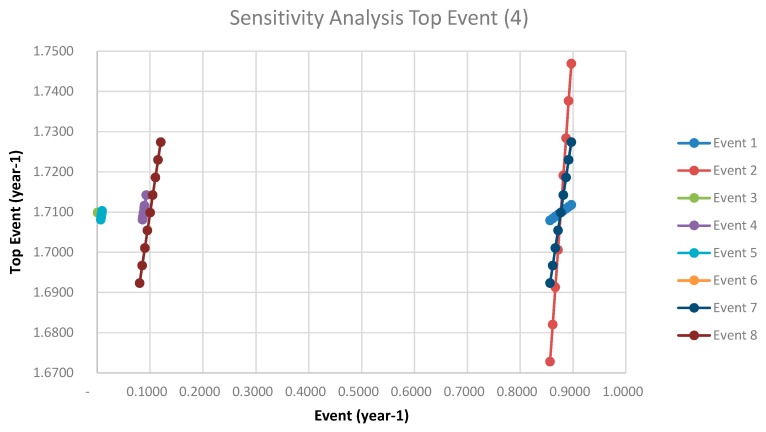
Sensitivity Analysis for the Top event (4).

## Appendix A. Historical Analysis of Accidents

**Table A1:** Analysis of Accidents.

Date	Location	Products Involved	Origin of Accident	Description
2010	Burosse-Mendousse (France) [44]	Oil	Explosion	Explosion of a tank of 1400 m^3^ containing crude oil. The roof was ejected several meters away and the tank’s base slightly lifted. The most probable ignition source is an electrostatic discharge.
2009	Bayamón (Puerto Rico) [43]	Gasoline, Diesel, Kerosene	Spill	In the plant of the Caribbean Petroleum Corporation (a storage, distribution, and fuel blending service) the failure of the sensor system for filling a gas tank caused a fuel spill that triggered a series of explosions and fires. The disaster affected 18 tanks, destroyed 50% of the plant, and caused considerable damage to the environment and the local area.
2007	Sløvâg (Norway) [44]	Gasoline	Fire	Accident took in the facilities of company Vest Tank AS, on the Sløvâg industrial area. The first explosion took place in a tank where the base–shell weld ruptured and the upper part of the tank was launched up in the air and landed in the north-eastern corner of Tank Farm II. Subsequent explosions and fires destroyed the other tank farm. There were no casualties in the accident. This accident occurred during purification of coker gasoline (reduction of the content of mercaptans). The investigation found that addition of hydrochloric acid during the process reduced the solubility of mercaptans in the solution, leading to the build-up of a flammable mixture. Air filter with activated carbon placed on the roof absorbed mercaptans, leading to a self-ignition and the explosion.
2006	Spoleto (Italy) [43]	Oil	Explosion	An explosion occurred at Umbria Oil plant near Spoleto, Italy, when five workers were welding a structure on the roofs of several tanks. Firstly, one tank containing raw pomace oil exploded, rising up of about 10 m. This first explosion led to a pool fire that spread in the tanks’ park. One hour later, two other tanks exploded, with rupture of the bottom welding, ejecting missiles of 10 tons 80 m away near warehouses storing by-products and packaging materials. Four workers lost their life in this accident.
2006	Partridge-Raleigh (USA) [44]	Petroleum	Explosion	The explosion at Partridge-Raleigh Oilfield was caused by sparks of the welding of pipes that joined tanks. Three workers died and other suffered serious injuries.
2005	Hertfordshire (England) [13,43]	Gasoline	Spill	In the storage terminal known as “Buncefield depot” 300 tons of gasoline overflowed in a storage tank because of a high-level device failure and the failure of safety device that close the filling valves and raise the alarm. Fire broke out when the gasoline vapour cloud ignited. The ignition source may have been a backup generator, or a spark produced by a vehicle. In total, 20 storage tanks (containing 13.5 million litres each) burned for several days.
2004	Skikda (Algeria) [42]	LNG	Explosion	The steam boiler of the LNG production plant exploded, triggering a second, more massive vapour-cloud explosion and fire. The explosions and fire destroyed a portion of the LNG plant and caused 27 deaths, 74 injuries, and material damage outside the plant’s boundaries.
2003	Puertollano (Spain) [10]	Naphta	Explosion	An explosion in a naphtha tank in the refinery resulted in an intense fire that spread to six other tanks containing 8600 m^3^ of gasoline.
2003	Oklahoma (USA) [44]	Diesel	Explosion	In a Conoco-Phillips plant a diesel tank exploded with 900 m^3^ of fuel, triggering a fire that involved three other liquid fuel storage tanks. The cause of the incident was the generation of a volatile mix inside the tank after it was emptied. The likely source of ignition was an electrical discharge from a nearby line.
2001	Kansas (USA) [10,12]	Crude petroleum	Fire	A worker who was checking the level of oil in a storage tank at night lit a match. The flame ignited vapours and caused a huge explosion.
2000	Hampshire (United Kingdom) [10]	Crude petroleum	Leak	A crack in the bottom of a storage tank of crude oil (caused by corrosion) caused a catastrophic spill of crude oil.
1997	Ashdod (Israel) [12]	Gasoil	Leak	In the tank farm of Ashdod Oil Refinery the explosion of a 15,000 m^2^ gasoil tank caused loss of one worker. The investigation concluded that a non-complete gasoil stripping with hydrogen at the exit of gasoil hydro treating unit caused penetration of hydrogen inside the tank. The source of ignition was most likely electrostatic spark initiated by synthetic rope used to get samples out the tank.
1995	Rouseville (USA) [44]	Wastewater Tank	Explosion	During a welding operation near the wastewater tank that contained a layer of flammable liquid, sparks ignited flammable vapours at openings in the tank. The deflagration caused the tank to fail at the bottom seam and shoot into the air. Five workers died and fire ignited other tanks and caused loud explosions.
1993	Port of Tarragona (Spain) [12]	Naphta, fuel oil and crude oil	Fire	A Danish petroleum tanker with 22,000 tons of naphtha on board collided with the REPSOL wharf in Tarragona during docking. The collision broke three pipes on the wharf containing naphtha, fuel oil, and crude oil—fire quickly broke out and produced a thick smoke. The combustion wastes contaminated nearby beaches. REPSOL estimated that damage to the wharf totalled the equivalent of €18 million.
1988	Santander (Spain) [12]	Diesel	Fire	A fire started during cleaning operations in an empty oil tank at a CAMPSA (now CLH) plant.
1987	Lyon (France) [10,12]	Gasoline and kerosene	Fire	A fire started in an enlarged Shell terminal holding up to 43,000 m^3^ of Class B oil products (gasoline and kerosene among others) and Class D products (asphalt). Nearly 7000 m^3^ of products were burned, two people dead, and 16 were seriously injured. The causes are unknown, although it is known that changes were being made to the wiring system.
1986	Thessaloniki (Greece) [41]	Fuel-oil	Leak	A fire caused by a fuel oil leak in an ESSO Pappas terminal set 10 of the 12 storage tanks ablaze. The fire lasted eight days, extended over 75% of the total area of the terminal, and destroyed the stationary fire-fighting system, as well as the systems controlling pumps and loading. The fire started during maintenance work after a leak in a tank went undetected.
1985	Port of Naples (Italy) [41,43]	Gasoline	Spill	At an AGIP plant a cloud of gasoline vapour exploded and damaged nearby houses. Windows broke up to 600 meters away. Tanks of gasoline, kerosene, and diesel were set on fire. The incident resulted in four deaths and 170 injuries. Twenty-four of the 32 storage tanks were affected. The probable cause was an accident when unloading a ship or a storage tank overflow.
1983	New Jersey (USA) [41]	Gasoline	Spill	An overfilled floating roof tank spilled 1300 barrels of gasoline. The resulting explosion destroyed two storage tanks and a neighbouring terminal. A cloud of vapour was blown to a nearby incinerator and set it on fire as well.
1979	Duisburg (Germany) [10,12,41]	Gasoil	Fire	In the river port area, a fire started in the storage area with 24 diesel and fuel oil storage tanks of between 1500 and 4700 m^3^ capacity. The accident occurred during the renovation of thermal insulation of the storage tanks.
1978	Stockton (USA) [10,12]	Gasoline and additives	Leak	A fire broke out in a plant with eight large tanks of petroleum products. Two of the gasoline storage tanks caught fire as well as various tanks containing additives. All stocks of foam within 90 km were used. The origin was a leak from a gasoline tank that produced a cloud of vapour which travelled about 220 m and came into contact with a water heater in a nearby yard.

LNG: Liquefied Natural Gas.

## Appendix C. Qualitative and Quantitative Top Events

**Table A2:** Qualitative evaluation of top event (2).

Top Event (2) Fuel Leak in Pipelines	
Equations System	Boolean Equation
A = B + C	A = 1 + 2 + 3 + (5 × 4) + (6 × 4) + (7 × 4)
B = 1 + 2 + 3	
C = D × 4	
D = 5 + 6 + 7	

**Table A3:** Top event failure frequencies (2).

Top Event (2) Fuel Leak in Pipelines		
Basic Event	Description	Failure Frequency (year^−1^)
1	Corrosion	4.4 × 10^−3^
2	Vehicles collision	8.8 × 10^−4^
3	Fatigue defect	8.8 × 10^−4^
4	Operator distracted	1.8 × 10^−3^
5	Pressure probe failure	4.1 × 10^−2^
6	Signal transmission failure	8.8 × 10^−1^
7	Valve shut-off response failure	2.2 × 10^−1^
D	Failure control leakage	1.14 × 10^0^
C	Undetected leak	2.0 × 10^−3^
B	Breakage caused by cracking	6.1 × 10^−3^
A = B + C	Top event (2)	8.1 × 10^−3^

**Table A4:** Qualitative evaluation of top event (3).

Top Event (3) Leak in Storage Tank	
Equations System	Boolean Equation
A = B + C	A = (2 × 1) + (3 × 1) + (4 × 1) + 5 + 6 + 7 + 8 + 9
B = D × 1	
C = E + F	
D = 2 + 3 + 4	
E = 5 + 6	
F = 7 + 8 + 9	

**Table A5:** Top event failure frequencies (3).

Top Event (3) Leak in Storage Tank		
Basic Event	Description	Failure Frequency (year^−1^)
1	Operator failure	8.8 × 10^−2^
2	Sensor level failure	4.1 × 10^−1^
3	Valve shut-off response failure	2.2 × 10^−1^
4	Acoustic signal failure	8.8 × 10^−2^
5	Reinforcement breaking	2.2 × 10^−3^
6	Tank breaking	2.2 × 10^−3^
7	Corrosion	4.4 × 10^−3^
8	Insufficient revisions	1.8 × 10^−3^
9	Operator failure	1.8 × 10^−3^
F	Crack formation leak	8.0 × 10^−3^
E	Catastrophic tank rupture	4.4 × 10^−3^
D	Level control failure	7.2 × 10^−1^
C	Loss of leak tightness	1.2 × 10^−2^
B	Overfilling	6.3 × 10^−2^
A = B + C	Top event (3)	7.5 × 10^−2^

**Table A6:** Qualitative evaluation of top event (4).

Top Event (4) Fuel Spill in Tank Truck Loading Area	
Equations System	Boolean Equation
A = B + C	A = (3 × 1) + (4 × 1) + (5 × 1) + (2 × 6) + (2 × 7) + (2 × 8)
B = D × 1	
C = 2 × E	
D = 3 + 4 + 5	
E = 6 + 7 + 8	

**Table A7:** Top event failure frequencies (4).

Top Event (4) Fuel Spill in Tank Truck Loading Area		
Basic Event	Description	Failure Frequency (year^−1^)
1	Operator failure	8.8 × 10^−1^
2	Hose incorrectly connected	8.8 × 10^−1^
3	Collision against hose	8.8 × 10^−4^
4	Hose defects due to misuse	8.8 × 10^−2^
5	Manufacturing effects	8.8 × 10^−3^
6	Incorrect alarm response	8.8 × 10^−1^
7	Acoustic signal failure	8.8 × 10^−1^
8	Manual shut-off valve sticking	1.0 × 10^−1^
E	Emergency action failure	1.86 × 10^0^
D	Broken Hose	9.7 × 10^−2^
C	Connection leak	1.63 × 10^0^
B	Leak caused by broken hose	8.5 × 10^−3^
A = B + C	Top event (4)	1.7 × 10^0^

## Appendix D. Sensitivity Analysis of Results

**Table A8:** Sensitivity Analysis for the Top event (2).

Top Event (2) Fuel Leak in Pipelines							
Equations System				A = B + C = (1 + 2 + 3 + (5 × 4) + (6 × 4) + (7 × 4)			
Event 1	B	C	A	Event 2	B	C	A
0.0064	0.0081	0.0020	0.0101	0.0011	0.0063	0.0020	0.0083
0.0059	0.0076	0.0020	0.0096	0.0010	0.0063	0.0020	0.0083
0.0054	0.0071	0.0020	0.0091	0.0010	0.0062	0.0020	0.0082
0.0049	0.0066	0.0020	0.0086	0.0009	0.0062	0.0020	0.0082
0.0044	0.0061	0.0020	0.0081	0.0009	0.0061	0.0020	0.0081
0.0039	0.0056	0.0020	0.0076	0.0008	0.0061	0.0020	0.0081
0.0034	0.0051	0.0020	0.0071	0.0008	0.0060	0.0020	0.0080
0.0029	0.0046	0.0020	0.0066	0.0007	0.0060	0.0020	0.0080
0.0024	0.0041	0.0020	0.0061	0.0007	0.0059	0.0020	0.0079
Event 3	B	C	A	Event 4	B	C	A
0.0011	0.0063	0.0020	0.0083	0.0020	0.0061	0.0022	0.0083
0.0010	0.0063	0.0020	0.0083	0.0019	0.0061	0.0022	0.0083
0.0010	0.0062	0.0020	0.0082	0.0019	0.0061	0.0021	0.0082
0.0009	0.0062	0.0020	0.0082	0.0018	0.0061	0.0021	0.0082
0.0009	0.0061	0.0020	0.0081	0.0018	0.0061	0.0020	0.0081
0.0008	0.0061	0.0020	0.0081	0.0017	0.0061	0.0019	0.0081
0.0008	0.0060	0.0020	0.0080	0.0017	0.0061	0.0019	0.0080
0.0007	0.0060	0.0020	0.0080	0.0016	0.0061	0.0018	0.0079
0.0007	0.0059	0.0020	0.0079	0.0016	0.0061	0.0018	0.0079
Event 5	B	C	A	Event 6	B	C	A
0.0432	0.0061	0.0020	0.0081	0.8966	0.0061	0.0020	0.0081
0.0427	0.0061	0.0020	0.0081	0.8916	0.0061	0.0020	0.0081
0.0422	0.0061	0.0020	0.0081	0.8866	0.0061	0.0020	0.0081
0.0417	0.0061	0.0020	0.0081	0.8816	0.0061	0.0020	0.0081
0.0412	0.0061	0.0020	0.0081	0.8766	0.0061	0.0020	0.0081
0.0407	0.0061	0.0020	0.0081	0.8716	0.0061	0.0020	0.0081
0.0402	0.0061	0.0020	0.0081	0.8666	0.0061	0.0020	0.0081
0.0397	0.0061	0.0020	0.0081	0.8616	0.0061	0.0020	0.0081
0.0392	0.0061	0.0020	0.0081	0.8566	0.0061	0.0020	0.0081
Event 7	B	C	A				
0.2212	0.0061	0.0020	0.0081				
0.2207	0.0061	0.0020	0.0081				
0.2202	0.0061	0.0020	0.0081				
0.2197	0.0061	0.0020	0.0081				
0.2192	0.0061	0.0020	0.0081				
0.2187	0.0061	0.0020	0.0081				
0.2182	0.0061	0.0020	0.0081				
0.2177	0.0061	0.0020	0.0081				
0.2172	0.0061	0.0020	0.0081				

**Table A9:** Sensitivity Analysis for the Top event (3).

Top Event (3) Leak in Storage Tank							
Equations System				A = B + C = (2 × 1) + (3 × 1) + (4 × 1) + 5 + 6 + 7 + 8 + 9			
Event 1	B	C	A	Event 2	B	C	A
0.1077	0.0774	0.0123	0.0896	0.4320	0.0648	0.0123	0.0770
0.1027	0.0738	0.0123	0.0860	0.4270	0.0643	0.0123	0.0766
0.0977	0.0702	0.0123	0.0825	0.4220	0.0639	0.0123	0.0761
0.0927	0.0666	0.0123	0.0789	0.4170	0.0634	0.0123	0.0757
0.0877	0.0630	0.0123	0.0753	0.4120	0.0630	0.0123	0.0753
0.0827	0.0594	0.0123	0.0717	0.4070	0.0626	0.0123	0.0748
0.0777	0.0558	0.0123	0.0681	0.4020	0.0621	0.0123	0.0744
0.0727	0.0522	0.0123	0.0645	0.3970	0.0617	0.0123	0.0740
0.0677	0.0486	0.0123	0.0609	0.3920	0.0613	0.0123	0.0735
Event 3	B	C	A	Event 4	B	C	A
0.2392	0.0648	0.0123	0.0770	0.0897	0.0632	0.0123	0.0754
0.2342	0.0643	0.0123	0.0766	0.0892	0.0631	0.0123	0.0754
0.2292	0.0639	0.0123	0.0761	0.0887	0.0631	0.0123	0.0754
0.2242	0.0634	0.0123	0.0757	0.0882	0.0631	0.0123	0.0753
0.2192	0.0630	0.0123	0.0753	0.0877	0.0630	0.0123	0.0753
0.2142	0.0626	0.0123	0.0748	0.0872	0.0630	0.0123	0.0752
0.2092	0.0621	0.0123	0.0744	0.0867	0.0629	0.0123	0.0752
0.2042	0.0617	0.0123	0.0740	0.0862	0.0629	0.0123	0.0751
0.1992	0.0613	0.0123	0.0735	0.0857	0.0628	0.0123	0.0751
Event 5	B	C	A	Event 6	B	C	A
0.00239	0.0630	0.01246	0.07547	0.0024	0.0630	0.01246	0.07547
0.00234	0.0630	0.01241	0.07542	0.0023	0.0630	0.01241	0.07542
0.00229	0.0630	0.01236	0.07537	0.0023	0.0630	0.01236	0.07537
0.00224	0.0630	0.01231	0.07532	0.0022	0.0630	0.01231	0.07532
0.00219	0.0630	0.01226	0.07527	0.0022	0.0630	0.01226	0.07527
0.00214	0.0630	0.01221	0.07522	0.0021	0.0630	0.01221	0.07522
0.00209	0.0630	0.01216	0.07517	0.0021	0.0630	0.01216	0.07517
0.00204	0.0630	0.01211	0.07512	0.0020	0.0630	0.01211	0.07512
0.00199	0.0630	0.01206	0.07507	0.0020	0.0630	0.01206	0.07507
Event 7	B	C	A	Event 8	B	C	A
0.00457	0.0630	0.01246	0.07547	0.00195	0.0630	0.01246	0.07547
0.00452	0.0630	0.01241	0.07542	0.00190	0.0630	0.01241	0.07542
0.00447	0.0630	0.01236	0.07537	0.00185	0.0630	0.01236	0.07537
0.00442	0.0630	0.01231	0.07532	0.00180	0.0630	0.01231	0.07532
0.00437	0.0630	0.01226	0.07527	0.00175	0.0630	0.01226	0.07527
0.00432	0.0630	0.01221	0.07522	0.00170	0.0630	0.01221	0.07522
0.00427	0.0630	0.01216	0.07517	0.00165	0.0630	0.01216	0.07517
0.00422	0.0630	0.01211	0.07512	0.00160	0.0630	0.01211	0.07512
0.00417	0.0630	0.01206	0.07507	0.00155	0.0630	0.01206	0.07507
Event 9	B	C	A				
0.00195	0.0630	0.01246	0.07547				
0.00190	0.0630	0.01241	0.07542				
0.00185	0.0630	0.01236	0.07537				
0.00180	0.0630	0.01231	0.07532				
0.00175	0.0630	0.01226	0.07527				
0.00170	0.0630	0.01221	0.07522				
0.00165	0.0630	0.01216	0.07517				
0.00160	0.0630	0.01211	0.07512				
0.00155	0.0630	0.01206	0.07507				

**Table A10:** Sensitivity Analysis for the Top event (4).

Top Event (4) Fuel Spill in Tank Truck Loading Area							
Equations System				A = B + C = (3 × 1) + (4 × 1) + (5 × 1) + (2 × 6) + (2 × 7) + (2 × 8)			
Event 1	B	C	A	Event 2	B	C	A
0.8966	0.0872	1.6246	1.7118	0.8966	0.0853	1.6616	1.7469
0.8916	0.0868	1.6246	1.7113	0.8916	0.0853	1.6524	1.7377
0.8866	0.0863	1.6246	1.7108	0.8866	0.0853	1.6431	1.7284
0.8816	0.0858	1.6246	1.7104	0.8816	0.0853	1.6338	1.7191
0.8766	0.0853	1.6246	1.7099	0.8766	0.0853	1.6246	1.7099
0.8716	0.0848	1.6246	1.7094	0.8716	0.0853	1.6153	1.7006
0.8666	0.0843	1.6246	1.7089	0.8666	0.0853	1.6060	1.6913
0.8616	0.0838	1.6246	1.7084	0.8616	0.0853	1.5968	1.6821
0.8566	0.0833	1.6246	1.7079	0.8566	0.0853	1.5875	1.6728
Event 3	B	C	A	Event 4	B	C	A
0.00090	0.08531	1.6246	1.70989	0.0897	0.0870	1.6246	1.7116
0.00089	0.08531	1.6246	1.70988	0.0892	0.0866	1.6246	1.7112
0.00089	0.08530	1.6246	1.70988	0.0887	0.0862	1.6246	1.7107
0.00093	0.08534	1.6246	1.70991	0.0927	0.0897	1.6246	1.7143
0.00088	0.08530	1.6246	1.70987	0.0877	0.0853	1.6246	1.7099
0.00087	0.08529	1.6246	1.70987	0.0872	0.0849	1.6246	1.7094
0.00087	0.08529	1.6246	1.70986	0.0867	0.0844	1.6246	1.7090
0.00086	0.08528	1.6246	1.70986	0.0862	0.0840	1.6246	1.7086
0.00086	0.08528	1.6246	1.70985	0.0857	0.0835	1.6246	1.7081
Event 5	B	C	A	Event 6	B	C	A
0.0090	0.0855	1.6246	1.7100	0.8966	0.0853	1.6421	1.7274
0.0089	0.0854	1.6246	1.7100	0.8916	0.0853	1.6377	1.7230
0.0089	0.0854	1.6246	1.7100	0.8866	0.0853	1.6333	1.7186
0.0093	0.0857	1.6246	1.7103	0.8816	0.0853	1.6290	1.7143
0.0088	0.0853	1.6246	1.7099	0.8766	0.0853	1.6246	1.7099
0.0083	0.0849	1.6246	1.7094	0.8716	0.0853	1.6202	1.7055
0.0078	0.0844	1.6246	1.7090	0.8666	0.0853	1.6158	1.7011
0.0073	0.0840	1.6246	1.7086	0.8616	0.0853	1.6114	1.6967
0.0068	0.0835	1.6246	1.7081	0.8566	0.0853	1.6070	1.6923
Event 7	B	C	A	Event 8	B	C	A
0.8966	0.0853	1.6421	1.7274	0.1201	0.0853	1.6421	1.7274
0.8916	0.0853	1.6377	1.7230	0.1151	0.0853	1.6377	1.7230
0.8866	0.0853	1.6333	1.7186	0.1101	0.0853	1.6333	1.7186
0.8816	0.0853	1.6290	1.7143	0.1051	0.0853	1.6290	1.7143
0.8766	0.0853	1.6246	1.7099	0.1001	0.0853	1.6246	1.7099
0.8716	0.0853	1.6202	1.7055	0.0951	0.0853	1.6202	1.7055
0.8666	0.0853	1.6158	1.7011	0.0901	0.0853	1.6158	1.7011
0.8616	0.0853	1.6114	1.6967	0.0851	0.0853	1.6114	1.6967
0.8566	0.0853	1.6070	1.6923	0.0801	0.0853	1.6070	1.6923
